# Silicon supply promotes differences in growth and C:N:P stoichiometry between bamboo and tree saplings

**DOI:** 10.1186/s12870-023-04443-0

**Published:** 2023-09-21

**Authors:** Xiaoyu Liu, Xinghao Tang, Zacchaeus G. Compson, Dongmei Huang, Guiwu Zou, Fenggang Luan, Qingni Song, Xiong Fang, Qingpei Yang, Jun Liu

**Affiliations:** 1 College of Forestry, Jiangxi Agricultural University, Nanchang, 330045 China; 2 Fujian Academy of Forestry, Fuzhou, 350002 China; 3 Department of Biological Sciences Advanced Environmental Research Institute, University of North Texas Denton, Denton, Texas USA; 4 School of Humanities and Public Administration, Jiangxi Agricultural University, Nanchang, 330045 China; 5 College of Land Resources and Environment, Jiangxi Agricultural University, Nanchang, 330045 China

**Keywords:** Silicon supply, Silicon accumulation, Photosynthetic capacity, C: N: P stoichiometry, Plant growth, Plant invasion, Bamboo

## Abstract

**Background:**

Si can be important for the growth, functioning, and stoichiometric regulation of nutrients for high-Si-accumulating bamboo. However, other trees do not actively take up dissolved silicic acid [Si(OH)_4_] from the soil, likely because they have fewer or no specific Si transporters in their roots. It is unclear what causes differential growth and C:N:P stoichiometry between bamboo and other trees across levels of Si supply.

**Results:**

Si supply increased the relative growth rate of height and basal diameter of bamboo saplings, likely by increasing its net photosynthetic rate and ratios of N:P. Moreover, a high concentration of Si supply decreased the ratio of C:Si in bamboo leaves due to a partial substitution of C with Si in organic compounds. We also found that there was a positive correlation between leaf Si concentration and its transpiration rate in tree saplings.

**Conclusions:**

We demonstrated that Si supply can decrease the ratio of C:Si in bamboo leaves and increase the ratio of N:P without altering nutrient status or the N:P ratio of tree saplings. Our findings provide experimental data to assess the different responses between bamboo and other trees in terms of growth, photosynthesis, and C:N:P stoichiometry. These results have implications for assessing the growth and competition between high-Si-accumulating bamboo and other plants when Si availability is altered in ecosystems during bamboo expansion.

**Supplementary Information:**

The online version contains supplementary material available at 10.1186/s12870-023-04443-0.

## Highlights

(1) Silicon (Si) supply improved the relative growth rate of height and basal diameter of bamboo saplings, which was in contrast to our findings for other tree saplings.

(2) Si supply altered the photosynthetic gas exchange properties of plants, which in turn altered Si absorption from soil via passively uptake controlled by leaf transpiration.

(3) The ratio of C:Si for all tissues in bamboo was much lower than other tree saplings. Specifically, Si supply decreased the ratio of C:Si in bamboo leaves due to a partial substitution of organic C compounds by Si.

**Supplementary Information:**

The online version contains supplementary material available at 10.1186/s12870-023-04443-0.

## Background

Silicon (Si), the second most abundant element in the Earth’s crust after oxygen, is released through the weathering of minerals, and some fractions contribute to biogeochemical cycles across long time scales [53]. Si can also regulate global CO_2_ concentrations through plant-induced silicate weathering [21, 53] and occluding carbon within phytoliths resistant to decomposition [6, 52]. Si accumulation in higher plants varies greatly among species due to their different Si uptake mechanisms [33]. Active, passive, and exclusive mechanisms if Si uptake in plant species, therefore, are classified as high-, intermediate-, and non-accumulators, respectively [8].

Moso bamboo (*Phyllostachys pubescens*) can actively uptake dissolved silicic acid [Si(OH)_4_] from soil via specific Si transporters in abundant fine roots, like rice and wheat ([17, 29, 32], which was different from trees. Most plants take up Si passively, influenced by transpiration [8]. In addition, bamboo has the potential to expand into neighboring (secondary) forest ecosystems and shift plant community composition due to its strong leptomorphic (running) rhizome system [11, 26]. Bamboo expansion is a widespread phenomenon in subtropical regions [11, 57]). For example, bamboo expansion can alter Si pools and fluxes in forest ecosystems, particularly Si availability in soil [29]. However, there is no consensus on the nutrient status and C:N:P stoichiometry of bamboo and other tree species, which hinders our ability to predict the growth and nutrient stoichiometry of bamboo and other trees, particularly when the available Si pool has been altered during bamboo expansion.

Generally, Si availability is determined by the concentration of non-crystalline Si in soil, which is dissolved relatively easily and can be easily absorbed and utilized by plants [22, 47]. Si supply could improve Si availability in agricultural and grassland ecosystems [18], potentially improving plant production. For example, Si was associated with high growth rates of crop plants [16, 24] and could therefore promote plant growth and increase the number of branches, resulting in higher production of aboveground biomass [27]. For example, Si improved dry biomass, nutrient accumulation, and grain yield in different crop systems [5, 19].

Si also plays an important role in improving plant photosynthesis and modifying C:N:P stoichiometry. For example, Si can mitigate some stressors in plants, promoting photosynthetic capacity and improving physiological processes due to nutrient accumulation and investment in structural strength and rigidity [36, 37]. Photosynthetic capacity is generally closely linked to leaf N and P content because both largely affect CO_2_ assimilation capacity of plants through altering the concentration of enzymes and ATP [24, 58]. Si has been reported to increase net photosynthetic rate and chlorophyll content [24, 56], would influence leaf N and P content. On the other hand, there was a partial substitution of organic C compounds by Si in some plants because Si can play a role similar to C in leaf structure, reflecting the variable ratio of C:Si, as Si supply altered Si absorption and accumulation into tissues of higher plants [20, 41], in fact, there would be cheaper energy cost in plant defense structure through Si rather than C [10, 51]. Further, some studies found that Si availability modifies nutrient use efficiency and C:N:P stoichiometry of some plants through expanding their root-to-canopy ratio [42, 48, 54]. Si deficiency in soil could even decrease plant growth and weaken its photosynthetic capacity. Consequently, increasing doses of Si can alter the stoichiometric composition of plants and, in turn, improve key physiological aspects, such as net photosynthetic rate and nutrients accumulation, leading to increased growth. Some experimental studies outline benefits for grass or crop plants from exogenous Si, such as increasing biomass production and modifying nutrient stoichiometry to favor plant growth [40, 48]. However, we still do not know whether Si is advantageous for the growth of bamboo and tree species, which are rarely studied in this context.

To explore the influences of Si availability on the growth, physiological characteristics, and C:N:P stoichiometry of bamboo and other trees, we selected one- year saplings of *P. pubescens*, *Phoebe bournei*, *Schima superba*, *Cunninghamia lanceolata*, and conducted a pot experiment using three different levels of Si application. We posed two a priori hypotheses. First, we hypothesized (H_1_) that there would be different growth responses to Si supply between bamboo and other trees due to their different mechanisms of Si uptake and transport. Specifically, we predicted that Si supply would promote the biomass production of bamboo, which would increase with its Si supply level, whereas trees would exhibit no response to Si supply due to their passive mechanisms of Si uptake determined by transpiration. Second, we hypothesized (H_2_) that Si supply would improve photosynthetic gas exchange and modify C:N:P stoichiometry of both bamboo and tree saplings, and that these effects would be stronger in bamboo. We posited that bamboo can take up dissolved silicic acid [Si(OH)_4_] from soil both actively and passively, unlike the passive mechanisms of Si uptake in other trees that are determined by transpiration. To test these hypotheses, we measured growth traits, photosynthetic gas exchange properties, and C:N:P stoichiometry of sapling responses to three levels Si supply and analyzed the impacts of Si supply on growth and nutrient status of bamboo and tree saplings. Our work builds a better framework for understanding differences in the responses of a range of physiologically different and commercially and ecologically important plant species to Si supply, which helps us predict differential performance between bamboo and other trees due to altered Si availability following bamboo expansion.

## Results

### Relative growth rate of height and basal diameter

**Fig. 1:**
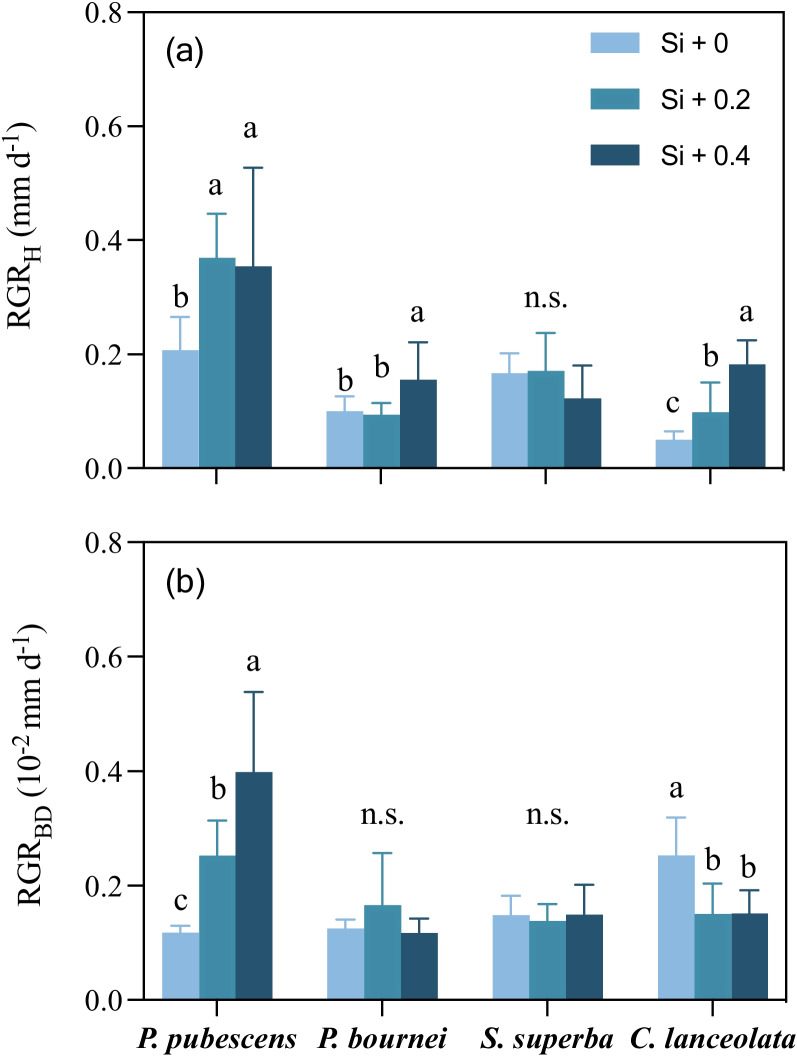
The relative growth rate of height (RGR_H_) and basal diameter (RGR_BD_) of *P. pubescens*, *P. bournei, S. superba*, and *C. lanceolata* among three levels of Si supply. Error bars depict means with standard errors (*n* = 8). Lower case letters denote significant differences among Si treatments for relative growth rate of height and basal diameter (*P* < 0.05)

Overall, for saplings except *S. superba*, the relative growth rate of height (RGR_H_) in the high concentration of Si supply (Si + 0.4) was higher than that in the control (Si + 0) (*F*_(2, 21)_ = 4.932, *P* = 0.018; *F*_(2, 21)_ = 5.063, *P* = 0.016;* F*_(2, 21)_ = 22.519, *P* = 0.000). For *P. pubescens*, both relative growth rate of basal diameter (RGR_BD_) in the high and middle concentration of Si supply (Si + 0.2 and Si + 0.4) were higher than that of the control (Si + 0) (*F*_(2, 21)_ = 20.089, *P* = 0.000), which was opposite of what was observed in *C. lanceolata* (*F*_(2, 21)_ = 9.535, *P* = 0.001) (Fig. 1).

### Photosynthetic gas exchange properties and water use efficiency

**Fig. 2:**
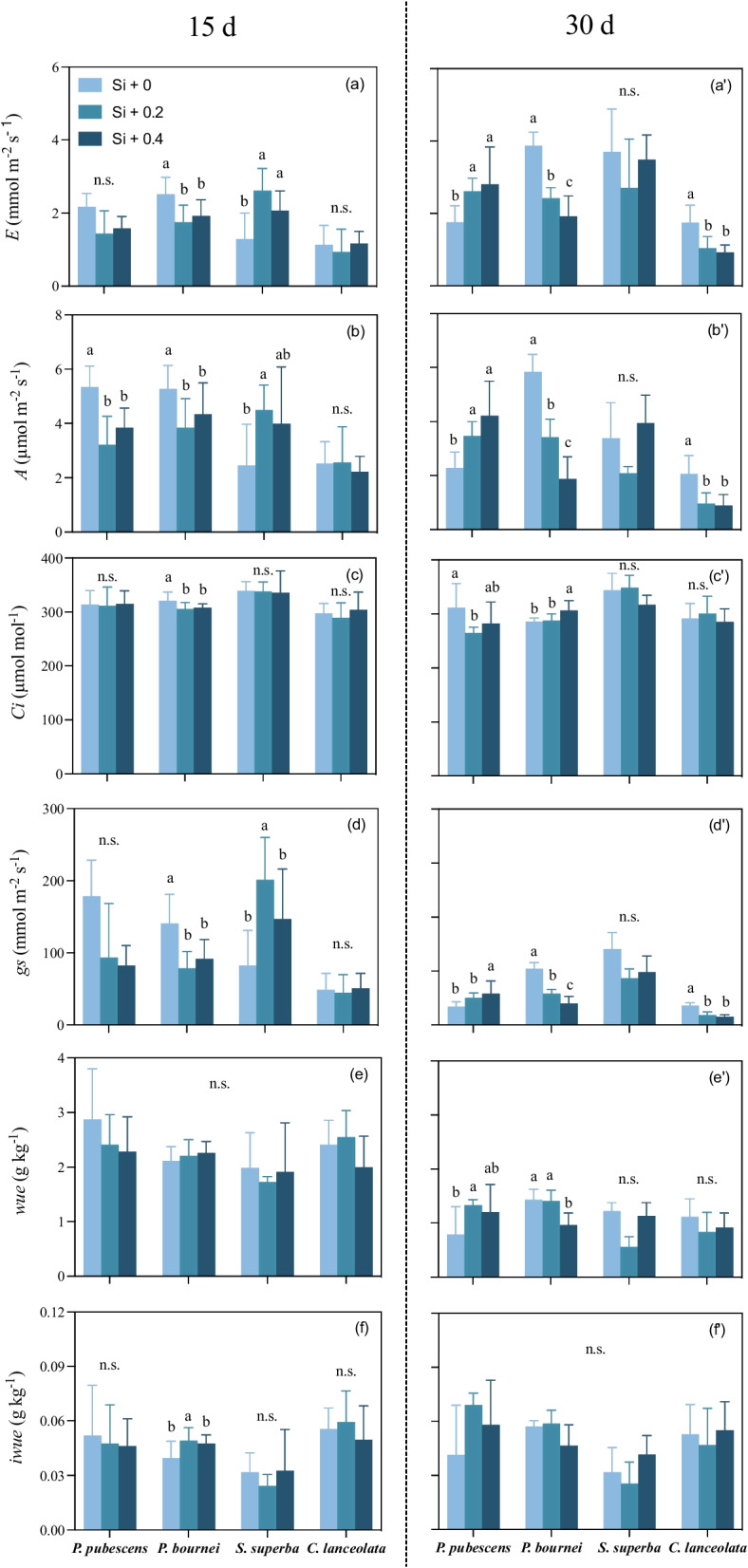
The photosynthetic gas exchange properties of plant saplings among three levels of Si supply: (a, a’) transpiration rate (*E*, mmol H_2_O m^−2^ s^−1^), (b, b’) net photosynthetic rate (*A*, µmol CO_2_ m^−1^ s^−1^), (c, c’) intercellular CO_2_ concentration (*Ci*, µmol CO_2_ m^−1^ s^−1^), (d, d’) stomatal conductance (*gs*, mmol H_2_O m^−2^ s^−1^), (e, e’) water use efficiency (*wue*, g kg^−1^), and (f, f’) intrinsic water use efficiency (*iwue*, g kg.^−1^). Error bars depict means with standard errors (*n* = 8). Lower case letters denote significant differences among three levels of Si supply for several photosynthetic gas exchange properties and water use efficiency (*P* < 0.05)

We observed different performances in photosynthetic gas exchange properties and water use efficiency of saplings at different times after Si supply. For example, 15 days after Si supply, there were no significant differences in stomatal conductance, transpiration rate, or intercellular CO_2_ concentrations of bamboo saplings among three levels of Si supply (*P* > 0.05), despite differences in net photosynthetic rate (*F*_(2, 19)_ = 9.801, *P* = 0.001). However, 30 days after Si supply, the transpiration rate, net photosynthetic rate, and stomatal conductance of bamboo in the high concentration Si supply (Si + 0.4) was higher than that in the control (Si + 0) (*F*_(2, 21)_ = 5.356, *P* = 0.013; *F*_(2, 21)_ = 6.248, *P* = 0.007; and* F*_(2, 21)_ = 5.205, *P* = 0.015, respectively), which was opposite to *C. lanceolata* (Si + 0) (*F*_(2, 20)_ = 11.705, *P* = 0.000;* F*_(2, 20)_ = 5.114, *P* = 0.016; and* F*_(2, 20)_ = 16.122, *P* = 0.000, respectively). In addition, there were different performances in photosynthetic gas exchange properties and water use efficiency for other tree saplings. For example, the transpiration rate, net photosynthetic rate, and stomatal conductance of *P. bournei* in the high concentration of Si supply (Si + 0.4) was lower than that in the control (Si + 0) (*P* < 0.05), which was opposite to *S. superba* at 15 days after Si supply (*F*_(2, 19)_ = 9.165, *P* = 0.002;* F*_(2, 19)_ = 3.655, *P* = 0.0045; and* F*_(2, 19)_ = 6.796, *P* = 0.006, respectively; Fig. 2a-b and a’-b’).

Water use efficiency (both *wue* and *iwue*) of *S. superba* and *C. lanceolata* did not differ among the three levels of Si supply (*P* > 0.05). However, *wue* of bamboo in the high concentration of Si supply (Si + 0.4) was higher than that in the control (Si + 0) (*F*_(2, 21)_ = 3.514, *P* = 0.048), which was different from *P. bournei*, particularly at 30 days after Si supply (*F*_(2, 19)_ = 4.768, *P* = 0.021; Fig. 2e, f and e’, f’).

### Ratios of C:Si, C:N, C:P, and N:P

**Fig. 3:**
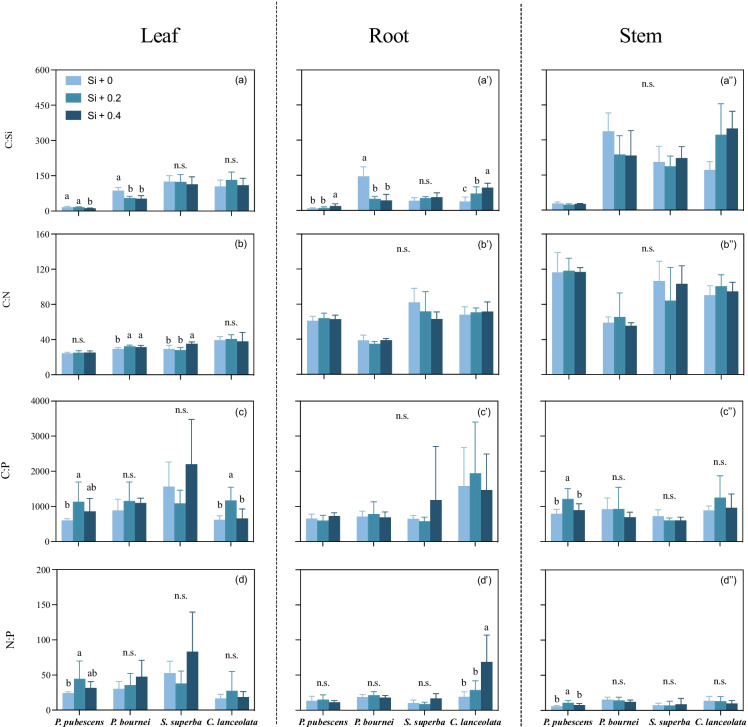
The ratios of C:Si, C:N, C:P and N:P in different tissues of *P. pubescens, P. bournei, S. superba* and *C. lanceolata* under three Si supply levels (*n* = 8): (**a**, **b**, **c**, **d**) leaf, (a’- d’) root and (a’’- d’’) stem. The data represent mean values and standard errors for the ratios of C:Si, C:N, C:P and N:P. Lowercase letters denote significant differences in the ratios of C:Si, C:N, C:P and N:P among varying Si supply levels within each tissue (*P* < 0.05)

Overall, the ratio of C:Si for all tissues in bamboo were much lower than other tree saplings (leaves: *F*_(3, 11)_ = 121.496, *P* < 0.001; roots:* F*_(3, 11)_ = 121.496, *P* < 0.001; and stems:* F*_(3, 11)_ = 121.496, *P* < 0.001; Fig. 3a-a’), with a higher concentration of Si and a lower concentration of C compared to other tree saplings (*F*_(3, 236)_ = 75.465, *P* < 0.001 and* F*_(3, 314)_ = 118.619, *P* < 0.001, respectively; Table S1). Additionally, we found no significant differences in the concentration of C, N, Si_SOL_, and Si_AMOR_ of soils among Si supply levels (Figure S2, all* P* > 0.05). When comparing Si supply treatments, bamboo leaves showed a lower ratio of C:Si for the high concentration of Si supply (Si + 0.4) compared to the ratio of C:Si in bamboo roots (*F*_(3, 11)_ = 121.496, *P* < 0.001 and *F*_(3, 11)_ = 121.496, *P* < 0.001, respectively; Fig. 3a). In addition, there were higher ratios of C:P and N:P in bamboo leaves for both Si supply treatments compared to the control treatment (Fig. 3c, d, *F*_(2, 21)_ = 3.639, *P* < 0.05 and *F*_(2, 21)_ = 3.478, *P* < 0.05, respectively). These results indicated that high concentrations of Si supply decreased the ratio of C:Si in bamboo leaves but increased the ratio of C:Si in bamboo roots, and Si supply disproportionately increased the ratios of C:P in *C. lanceolata* leaves and N:P in *C. lanceolata* roots. For leaves and roots of *P. bournei*, the ratio of C:Si in the control was higher than in both Si supply treatments (*F*_(2, 21)_ = 21.953, *P* < 0.001 and *F*_(2, 21)_ = 33.282, *P* < 0.001 in leaves and roots, respectively; Fig. 3a, a’); this pattern differed from the ratio of C:Si in *C. lanceolata* roots, which increased with increasing levels of Si supply (*F*_(2, 21)_ = 13.335, *P* < 0.001; Fig. 3a’). There were no significant differences in ratios of C:Si and C:N for sapling stems among three levels of Si supply (all *P* > 0.05), while the ratios of C:P and N:P in bamboo stems for the middle concentration of Si supply (Si + 0.2) were higher than the ratios in the control and high-Si-supply treatments (Si + 0 and Si + 0.4) (*F*_(2, 21)_ = 8.043, *P* = 0.003 and *F*_(2, 21)_ = 9.323, *P* = 0.001, respectively; Fig. 3a’’- d’’).

### Relationships of response variables with leaf traits

**Fig. 4:**
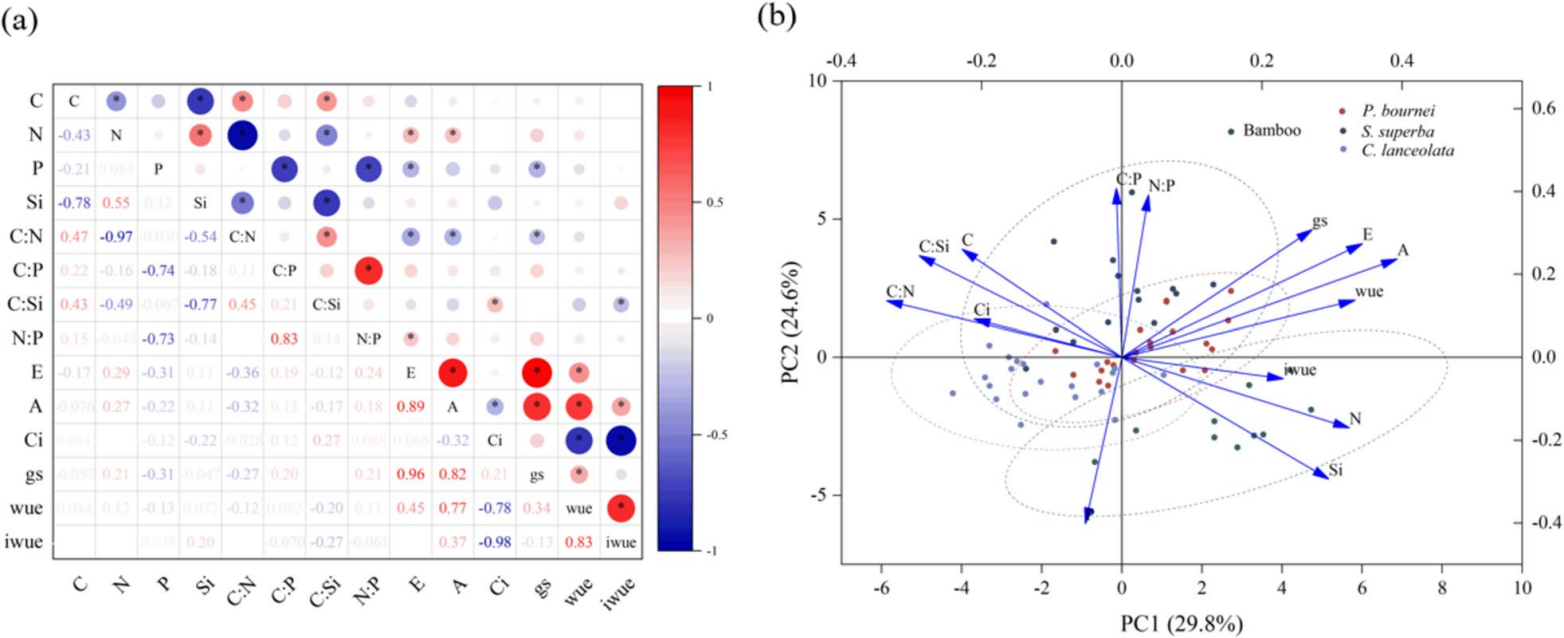
Correlations between leaf traits from bamboo and other tree saplings. **a** Semi-matrix of Pearson’s correlation coefficients between 14 leaf traits. The red and bule bubbles represent positive and negative correlation and to right represent negative correlation; the values represent the correlation coefficient. *P < 0.05. **b** Trait dimensions from principal component (PCA) analysis. Leaf traits of bamboo and other tree saplings are indicated combinations for PC1 vs PC2 and dotted with four colors (Fig. 5). PC1 explains 29.8% of trait variation and PC2 explains 24.6% of trait variation. The leaf variables considered were the concentration of N, C, Si, P, the ratios of C:Si, C:N, C:P and N:P and transpiration rate (*E*), net photosynthetic rate (*A*), stomatal conductance (*gs*), intercellular CO_2_ concentration (*Ci*), water use efficiency (*wue*) and intrinsic water use efficiency (*iwue*)

Overall, PCA axis 1 and 2 explained 29.8% and 24.6% of leaf trait variation, respectively. For PCA axis 1, which captured key photosynthetic gas exchange properties (i.e.* A*,* E*, *wue*), the ratio of C:N and the concentration of N and Si were the main variables with high loading factors. For axis 2 of the PCA, which captured key P variables, the concentration of P and ratios of C:P and N:P were the variables that had the high loading factors. Bamboo and tree saplings clearly divided into 2 groups. Bamboo was located in the positive sector of axis 1 and negative sector of axis 2, with a higher concentration of Si, which was opposite to the patterns observed in the other tree saplings with higher concentrations of C (Fig. 4b).

**Fig. 5:**
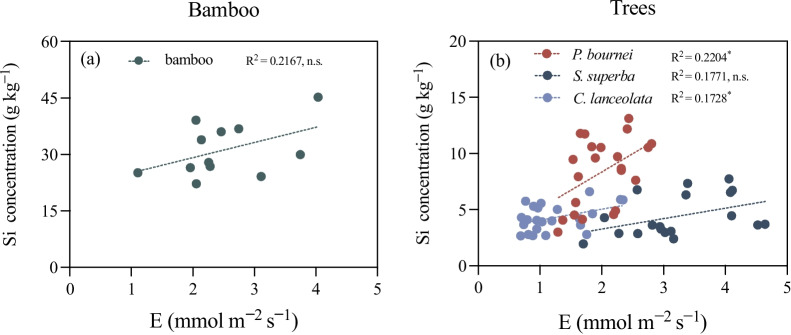
The linear relationships (Pearson’s R^2^; asterisks designate *P* < 0.05) between the Si concentration (g kg^−1^) of leaf and transpiration rate (*E*; mmol H_2_O m^−2^s^−1^) in (**a**) bamboo and (**b**) other tree saplings, including* P. bournei, S. superba *and *C. lanceolata*. The data represent single values for leaf concentration of Si and respective transpiration rates. Note the difference in scale of y-axes for the Si concentration of leaves between bamboo and tree saplings

Leaf concentration of Si was negatively correlated to the concentration of C and the ratio of C:N, and positively correlated with the concentration of N of saplings (Fig. 4, all *P* < 0.05). There was also a significant positive correlation between transpiration rate (*E*) and net photosynthetic rate (*A*), as well as stomatal conductance (*gs*) (*P* < 0.05; Fig. 4). Water use efficiency (i.e., wue and iwue) was positively correlated to net photosynthetic rate (**A**), but negatively correlated to intercellular CO_2_ concentration (*Ci*) (*P* < 0.05; Fig. 4). Moreover, we found that there was a positive relationship between the leaf Si concentration and its transpiration rate in saplings, particularly in *P. bournei* and *C. lanceolata* (R^2^ = 0.220, *P* = 0.028 and R^2^ = 0.173, *P* = 0.049, respectively; Fig. 5).

## Discussion

### Si modification of relative growth rates of plant saplings

Overall, Si supply increased bamboo’s relative growth rate of basal diameter (RGR_BD_), which was opposite to the pattern exhibited by *C. lanceolata*, supporting H_1_. The high-concentration Si supply also improved the relative growth rate of height (RGR_H_) for all saplings, except *S. superba* (Fig. 1). Other studies found that Si supply could promote the growth of plants by increasing their root-to-canopy ratios [4, 44], as seen in some Si accumulating plants, such as *P. australis* and *T. aestivum*. Liu et al. [27] found that Si promoted aboveground biomass by increasing plant height growth and its branching ability. In addition, a meta-analysis showed that Si supply increased the dry weight of stressed plants, but high-Si-accumulating Poaceae plants did not show stronger responses compared to other families, contrary to expectations [7]. For example, Si was acquired as a nutrient through specific Si transporters in roots of high Si-accumulating Poaceae, which contributed to alleviating the impacts of biotic and abiotic stresses, thereby increasing the biomass and production of Si-accumulating plants specifically [1, 13, 33].

### Si regulation of photosynthetic gas exchange properties

High-concentration Si supply increased the transpiration rate, net photosynthetic rate, and stomatal conductance of bamboo, particularly 30 days after application of the Si supply, which was consistent with H_2_ and other studies that examined different Si-accumulating plants [23, 55]. It has been reported that Si decreased the angle between the stem and plant leaves, thereby increasing light transmittance and improving its photosynthetic efficiency [25, 45]. Si is known to increase the biosynthesis of photosynthetic pigments, such as chlorophylls and carotenoids [14, 35]. Furthermore, Si supply could alter the photosynthetic gas exchange properties of plants, which in turn could increase or decrease Si absorption from soil because most plants take up [Si(OH)_4_] passively from soil, a process influenced by transpiration [39]. We found that there was a weak, positive correlation between leaf Si concentration and its transpiration rate in some saplings (i.e., *P. bournei* and *C. lanceolata*), indicating that plant Si concentration might be influenced by transpiration rate instead of other plant traits, as evidenced by this phenomenon occurring in some non-Si accumulating plants; however, given that these relationships were generally weak (i.e., all R^2^ < 0.23), it is likely that other plant traits also contributed plant Si concentrations, including potentially important variables that were not measured in our study, especially given the low amount of variation explained by our PCA results. Future studies should consider measuring other possible plant traits (e.g., leaf habits and life span) and environmental variables associated with plant Si concentration (e.g., soil parent material and weathering). Furthermore, we observed species-specificity in plant Si concentrations in our study. The capacity of plants for silica absorption varies considerably according to genotype and environment [46]. Specifically, various active and passive mechanisms of Si uptake have been shown to be integrated into plants, such as mechanisms that interact with Si uptake of Si-accumulating plants via specific Si transporters in roots [8].

### Si modification of C:N:P stoichiometry in plant saplings

Generally, Si supply increases Si availability in soils, but in this study there was no significant difference in the concentration of soluble Si (Si_SOL_) in pot soil among Si supply levels (Figure S2). This was likely due, in part, to the fact that silicic acid was quickly taken up by roots of plants actively via specific transporters or passively through transpiration [38]. Additionally, amorphous Si is unstable and easily transformed to silicic acid to keep a dynamic balance with other forms of Si in soil [49], contributing to relatively unchanged concentrations of Si_SOL_.

Overall, leaf concentrations of Si were negatively correlated to the concentration of C and the ratio of C:N, and positively correlated to the concentration of N among 14 leaf traits for all saplings (Fig. 4). Specifically, we found that the ratio of C:Si for all tissues in bamboo was much lower than for other tree saplings, with relatively higher concentrations of Si and lower concentrations of C (Table S1). Bamboo can actively uptake dissolved silicic acid [Si(OH)_4_] from soil via abundant fine roots with specific Si transporters [28, 29]. The structural role played by Si is related to its incorporation into cell walls, such as phytoliths, which contribute to defense against herbivores and pathogens [33, 38]. High-concentration Si supply decreased the ratio of C:Si in bamboo leaves but increased the ratios of C:P and N:P (Fig. 3). These results were probably due to a partial substitution of organic C with Si in aboveground tissues of *P. pubescens*, reflecting a switch of *P. pubescens* to an inexpensive defense mechanism when sufficient Si was available in the environment [20, 48]. This would be advantageous for the plant because Si deposits, including phytholiths, are energetically cheaper to form and can confer similar defenses to alleviate biotic or abiotic stress for plants [51]. Thus, we suspect that there were more competitive advantages in bamboo due to Si defense compared to other trees during bamboo expansion.

Si supply increased the leaf ratio of C:N in *P. bournei* and *S. superba* by altering their concentration of C and N, but it did not affect the ratio of N:P in plant saplings, except for bamboo. We speculate that tree saplings could not regulate nutrient status or modify the ratio of N:P in elevated Si treatments, but that this stoichiometric regulation could occur in high Si-accumulating bamboo. Leaf photosynthetic capacity of plants could influence their concentrations of N and P, which are therefore unlikely to be affected by Si supply in the short term [24, 58]. Phosphate uptake and distribution in plants is under genetic control [3], whereas uptake of Si is either passively or actively mediated by special groups of aquaporins, with subsequent genetic control [33, 34, 48].

## Conclusion

Si can be important for the growth, functioning, and stoichiometric regulation of nutrients for high-Si-accumulating bamboo. Si supply increased the relative growth rate of height and basal diameter of bamboo saplings. The ratio of C:Si for all tissues in bamboo were much lower than other tree saplings, and high-concentration Si supply decreased the ratio of C:Si in bamboo leaves due to a partial substitution of organic C compounds by Si in aboveground tissues and bamboo’s active Si uptake mechanisms. Si supply had minimal effects on nutrient status or the N:P ratio in tree saplings, but clear changes did occur in the N:P ratio observed in high-Si-accumulating bamboo under elevated Si conditions. Collectively, our findings provide experimental data to assess the different responses of bamboo and other trees in terms of growth, photosynthesis, and C:N:P stoichiometry under varying Si supply levels. These results suggest that the differential performance between bamboo and other trees is due to altered Si availability in the ecosystem during bamboo expansion.

## Methods

### Plant material, growth conditions, and experimental design

A pot experiment was conducted from March to November 2019 at Fujian Academy of Forestry Sciences, Fuzhou, Fujian, China (26.15°N 119.29° E). The mean air temperature and precipitation range are 20.3 °C and 1,558 ~ 1,800 mm, respectively. The light exposure was 6000–140000 Lux from morning to midday. We selected four species, *Phyllostachys pubescens*, *Phoebe bournei*, *Schima superba*, *Cunninghamia lanceolata*, which were identified formally by Q. Yang according to Flora of China (Flora of China, 2018). All saplings were one-year old and developed from seeds (Fujian Academy of Forestry Sciences, Fuzhou, China), which mean height and diameter were 40 cm and 5 mm respectively. These saplings represented the dominant species associated with bamboo expansion in subtropical forests. We calculated the mean weight of each species before planting (*n* = 8) in plastic pots (D: 20 cm, H: 35 cm) in January 2019, and filled with 3.5 kg homogenized natural soil per pot.

All saplings were grown outside and experienced natural and environmental conditions, with the same temperature and light exposure. These saplings were treated with three levels of Si supply in June: Si + 0 (control), Si + 0.2, and Si + 0.4 g per pot (equal to 7 g/m^2^ and 14 g/m^2^) [2, 27] (Fig. S1). A solution of H_2_SiO_3_ (Silicic acid, Sinopharm Chemical Reagent Co., Ltd.) neutralized with diluted H_2_SO_4_ was used as a silicon source [9, 43]. We frequently shuffled the position of the potted plants to prevent rooting underground, and reduce variability in light and humidity associated with pot position. We destructively sampled entire plants (leaves, stems, and roots) at 15 and 30 days after Si supply and simultaneously measured photosynthetic gas exchange properties (*n* = 8). All sampled saplings were collected, washed, and separated into leaves, stems, and roots, and then fully dried (65 °C) for 3 days. All plant samples were homogenized in a ball-mill (JX-2010, Shanghai, China) in preparation for chemical analyses. To determine soil chemical properties, we mixed soil in each plastic pot (about 300–400 g wet weight per sample). Then, soil samples were pooled, air-dried, and ground through a 2-mm sieve (*n* = 192 soil samples total).

### Relative growth rate of height and basal diameter

We measured the height and basal diameter of each sapling monthly. The relative growth rates of height and basal diameter were calculated as follows:

1 $${\mathrm{RGR}}_{\mathrm{H}} = \frac{\mathrm{ln}({\mathrm{H}}_{2})-\mathrm{ln}{(\mathrm{H}}_{1})}{{\mathrm{t}}_{2}-{\mathrm{t}}_{1}}$$

where RGR_H_ were the relative growth rates of height of saplings. H_1_ and H_2_ were the height (cm) of a given sapling measured twice at different times, t_1_ and t_2_ were measured in days (d).

2 $${\mathrm{RGR}}_{\mathrm{BD}} = \frac{\mathrm{ln}({\mathrm{BD}}_{2})-\mathrm{ln}{(\mathrm{BD}}_{1})}{{\mathrm{t}}_{2}-{\mathrm{t}}_{1}}$$

where RGR_BD_ were the relative growth rates of basal diameter of saplings, respectively, BD_1_ and BD_2_ were the basal diameter (mm) of a given sapling measured twice at different times, t_1_ and t_2_, measured in days (d).

### Photosynthetic gas exchange measurements

Photosynthetic traits, such as net photosynthetic CO_2_ assimilation (*A*), transpiration rate (*E*), stomatal conductance (*g*_s_), and internal CO_2_ concentrations (*Ci*), were observed approximately 15 and 30 days after Si supplementation, using an Li-COR 6800 portable photosynthesis system (Li-COR Biosciences, Lincoln, NE, USA). For each Si supply treatment, photosynthetic traits were measured between 09:00–11:00 a.m. (solar time) on treated and normal plants (*n* = 8), which is when *A* was at its maximum and temperature was suitable inside the growth chamber. Photosynthetic photon flux density (PPFD) was set at 1,000 μmol m^−2^ s^−1^, leaf temperature was 25 °C, and CO_2_ concentration was 400 μmol mol^−1^. Water use efficiency (*wue*), the carbon uptake per unit of water consumption. The response of *wue* at the leaf level is directly related to the physiological processes controlling the gradients of CO_2_ and H_2_O, including water use efficiency (*wue*) and intrinsic water use efficiency (*iwue*) were calculated as follows:

3 $$wue = \frac{A}{E}$$

4 $$iwue = \frac{A}{{g}_{s}}$$

### Chemical analyses

To extract total Si (Si_TOT_) from soil and plant tissues, 100 mg of dried leaf powder from each sample was digested using sodium hydroxide fusion at 650 °C in a muffle furnace following an established hydrochloric acid (1:1) dissolution method [30, 43]. Prior to analysis, the digested extract was diluted with deionized water up to 250 mL (final volume). Four non-crystalline Si fractions were extracted sequentially using the following procedure. (1) We added 30 mL of 1 M acid and Na acetate buffer solution at pH 4.0 to 0.75 g soil in a 50 mL centrifuge tube, agitated the sample for 24 h, and centrifuged the sample to extract the soluble and exchangeable Si (Si_SOL_ / Acid Na-acetate-Si), which is the most easily absorbed form of Si utilized by plants [22, 47], (2) We then added 5 mL H_2_O_2_ (30%) to the residue from step 1, heated the sample in a heated water-bath at 85 ± 2 °C for 1 h, added 30 mL of 1 M acid and Na acetate buffer solution at pH 4.0, agitated the sample for 24 h, and centrifuged to extract organic matter bound Si (Si_ORG_ / H_2_O_2_-Si). (3) Next, we added 30 mL 0.5 M NH_2_OH-HCl to the residue from step 2, wrapped the tubes in foil, agitated the sample for 24 h, and centrifuged to extract pedogenic oxides/hydroxides for chemisorbed Si (Si_SORB_ / NH_2_OH-HCl-Si). (4) Finally, we added 30 mL 0.5 M NaOH to the residue from step 3, treated the sample in an ultrasonic bath for 1 h, agitated for 24 h, and centrifuged to extract amorphous Si (Si_AMOR_/ NaOH-Si). All Si extractions (including extractions for Si_TOT_ [soil and plant], Si_SOL_, Si_ORG_, Si_SORB_, and Si_AMOR_ [soil]) were then analyzed using spectroscopy with the silicon–molybdenum blue colorimetric method and a molybdate–ascorbic acid procedure at a pH of 1.2 ~ 1.3 using an ultraviolet visible spectrophotometer (680 nm, UV-5100) [15, 52] calibrated with a standard solution (5 mg L^−1^ SiO_4_^2−^). All Si measurements were run in duplicate and averaged (variation among replicates < 5%).

All plant and soil samples (i.e., leaves, stems, roots, and soil) were analyzed for total N and total carbon (C) using a dry combustion analyzer (Vario EL, Elementar Analysensysteme GmbH, Langenselbold, Germany) with acetanilide as an external standard. All C/N measurements were run in duplicate and averaged because average deviations of replicates were 1.1% for N and 0.2% for C concentrations. All data are presented as the arithmetic mean values.

To extract P in samples, 150 mg of dried plant powder from each sample was digested using H_2_SO_4_ and H_2_O_2_ at 350 °C for half an hour using a microwave digestion system (JKXZ06-8B, China), followed by a deionized water wash (up to 100 mL final volume). Prior to analysis, all digested samples were then determined by spectroscopy using the molybdenum antimony spectroscopic method followed by a molybdate–ascorbic acid procedure using an ultraviolet visible spectrophotometer (880nm, UV-5100) calibrated with a standard solution (2.5 mg L^−1^ KH_2_PO_4_) [30]. All P measurements were run in duplicate and averaged (variation among replicates < 5%).

### Statistical analysis

We determined concentrations Si, C, N and P, the ratios of C:Si, C:N, C:P and N:P in plant tissues (including leaf, root and stem), and transpiration rate (*E*), net photosynthetic rate (*A*), stomatal conductance (*gs*), intercellular CO_2_ concentration (*Ci*), water use efficiency (*wue*), and intrinsic water use efficiency (*iwue*) of all saplings. We then compared these sapling traits among three levels of Si supply. Differences among levels of Si concentration for each response variable [including relative growth rate of height (RGR_H_) and basal diameter (RGR_BD_), photosynthetic gas exchange properties, and ratios of C:Si, C:N, C:P and N:P)] were determined using analysis of variance (ANOVA) and a protected least significant difference (LSD) test [12].

Principal components analysis (PCA), as a dimensionality reduction technique, is a powerful multivariate analysis technique for many ecological applications [31, 50]. Here, PCA was used to analyze trait covariation and a semi-matrix of Pearson’s correlation analysis to determine relationships between 14 leaf traits among all saplings. We also used correlation analysis and Pearson’s r to determine linear relationships between the leaf Si concentration and its transpiration rate, with the effect of the species coded as random effects. For all tests, the criterion for significant differences was α = 0.05. All statistical analyses were performed using SPSS (IBM, SPSS 24.0., Chicago, USA.), and graphs were created using Prism (GraphPad Prism 9.0, China).

### Supplementary Information

**Additional file 1: Table S1. **The concentrations of Si, C, N, and P in different tissues of *P. pubescens, P. bournei, S. superba, *and *C. lanceolata* under three Si supply levels (*n* = 8)*.*

**Additional file 2: Figure S1. **Conceptual diagram of the pot experiment, including three levels of Si supply: 0 (control), 0.2 g, and 0.4 g Si addition per pot. **Figure S2. **The concentration of nitrogen (N, g kg^−1^), carbon (C, g kg^−1^), soluble silicon (Si_SOL_, g kg^−1^), and amorphous silicon (Si_AMOR_, g kg^−1^) in soils under three levels of silicon supply. Error bars depict means with standard errors (*n* = 8). Different letters above bars reflect significant differences among groups (*P* < 0.05).

## Data Availability

All data generated or analyzed during this study are included in this published article.
