# Supplementary material for: Silicon supply promotes differences in growth and C:N:P stoichiometry between bamboo and tree saplings
Source: BMC Plant Biol. 2023 Sep 21;23:443. doi: 10.1186/s12870-023-04443-0 (PMC10512617; doi:10.1186/s12870-023-04443-0)
Supplement: Supplementary file 1 — Additional file 1: Table S1. The concentrations of Si, C, N, and P in different tissues of P. pubescens, P. bournei, S. superba, and C. lanceolata under three Si supply levels (n = 8). [file 12870_2023_4443_MOESM1_ESM.docx]

# Supplemental Materials

**Table S1. The concentrations of Si, C, N, and P in different tissues of *P. pubescens, P. bournei, S. superba,* and *C. lanceolata* under** **three Si supply levels (*n* = 8)*.*** The data represent mean values for the concentrations of Si, C, N, and P. Lowercase letters denote significant differences in concentration of Si, C, N, P among varying silicon supply levels (one-way ANOVA, α = 0.05) within species and tissue types. Significant differences are shown in bold.

| Elements | Si treatment | *P. pubescens* | | |  | *P. bournei* | | |  | *S. superba* | | |  | *C. lanceolata* | | |
| --- | --- | --- | --- | --- | --- | --- | --- | --- | --- | --- | --- | --- | --- | --- | --- | --- |
|  |  | Leaf | Stem | Root |  | Leaf | Stem | Root |  | Leaf | Stem | Root |  | Leaf | Stem | Root |
| Si (g kg^-1^) | Si + 0 | **28.0^b^** | **17.2^b^** | **30.9^b^** |  | **6.2^b^** | 1.7 | **5.3^b^** |  | 4.2 | 2.7 | 13.1 |  | 4.8 | **2.9^a^** | **14.8^a^** |
|  | Si + 0.2 | **28.7^b^** | **19.2^ab^** | **36.3^ab^** |  | **10.0^a^** | 2.2 | **10.0^ab^** |  | 4.8 | 2.6 | 9.5 |  | 3.8 | **1.8^b^** | **7.7^b^** |
|  | Si + 0.4 | **37.0^a^** | **21.8^a^** | **44.8^a^** |  | **9.3^a^** | 2.4 | **16.9^a^** |  | 4.7 | 2.3 | 9.7 |  | 6.6 | **1.4^b^** | **5.7^b^** |
| C (%) | Si + 0 | **42.4^a^** | **45.7^a^** | **37.1^a^** |  | 46.9 | 45.3 | 45.2 |  | 46.0 | 46.4 | 44.9 |  | 45.4 | 45.7 | 44.7 |
|  | Si + 0.2 | **41.5^ab^** | **42.7^b^** | **37.2^a^** |  | 46.7 | 45.2 | 45.6 |  | 46.5 | 45.5 | 44.8 |  | 45.8 | 45.6 | 45.2 |
|  | Si + 0.4 | **41.5^b^** | **42.7^b^** | **35.1^b^** |  | 46.7 | 45.8 | 45.3 |  | 46.4 | 45.2 | 44.6 |  | 45.3 | 45.6 | 45.6 |
| N (%) | Si + 0 | 1.82 | 0.39 | 0.63 |  | **1.75^a^** | 0.42 | **1.30^ab^** |  | **1.68^a^** | 0.52 | 0.67 |  | 1.22 | 0.51 | 0.72 |
|  | Si + 0.2 | 1.78 | 0.43 | 0.60 |  | **1.52^b^** | 0.45 | **1.46^a^** |  | **1.76^a^** | 0.55 | 0.72 |  | 1.26 | 0.54 | 0.78 |
|  | Si + 0.4 | 1.79 | 0.41 | 0.55 |  | **1.49^b^** | 0.41 | **1.22^b^** |  | **1.32^b^** | 0.51 | 0.80 |  | 1.31 | 0.51 | 0.71 |
| P (g kg^-1^) | Si + 0 | 0.79 | 0.71 | 0.52 |  | 0.62 | 0.71 | 0.71 |  | 0.69 | 0.36 | 0.69 |  | 0.74 | 0.51 | 0.40 |
|  | Si + 0.2 | 0.57 | 0.35 | 0.53 |  | 0.52 | 0.35 | 0.69 |  | 0.73 | 0.52 | 0.84 |  | 0.66 | 0.46 | 0.39 |
|  | Si + 0.4 | 0.62 | 0.61 | 0.53 |  | 0.48 | 0.61 | 0.71 |  | 0.71 | 0.28 | 0.70 |  | 0.78 | 0.59 | 0.36 |
