# Supplementary material for: Silicon supply promotes differences in growth and C:N:P stoichiometry between bamboo and tree saplings
Source: BMC Plant Biol. 2023 Sep 21;23:443. doi: 10.1186/s12870-023-04443-0 (PMC10512617; doi:10.1186/s12870-023-04443-0)
Supplement: Supplementary file 2 — Additional file 2: Figure S1. Conceptual diagram of the pot experiment, including three levels of Si supply: 0 (control), 0.2 g, and 0.4 g Si addition per pot. Figure S2. The concentration of nitrogen (N, g kg−1), carbon (C, g kg−1), soluble silicon (SiSOL, g kg−1), and amorphous silicon (SiAMOR, g kg−1) in soils under three levels of silicon supply. Error bars depict means with standard errors (n = 8). Different letters above bars reflect significant differences among groups (P < 0.05). [file 12870_2023_4443_MOESM2_ESM.docx]

| 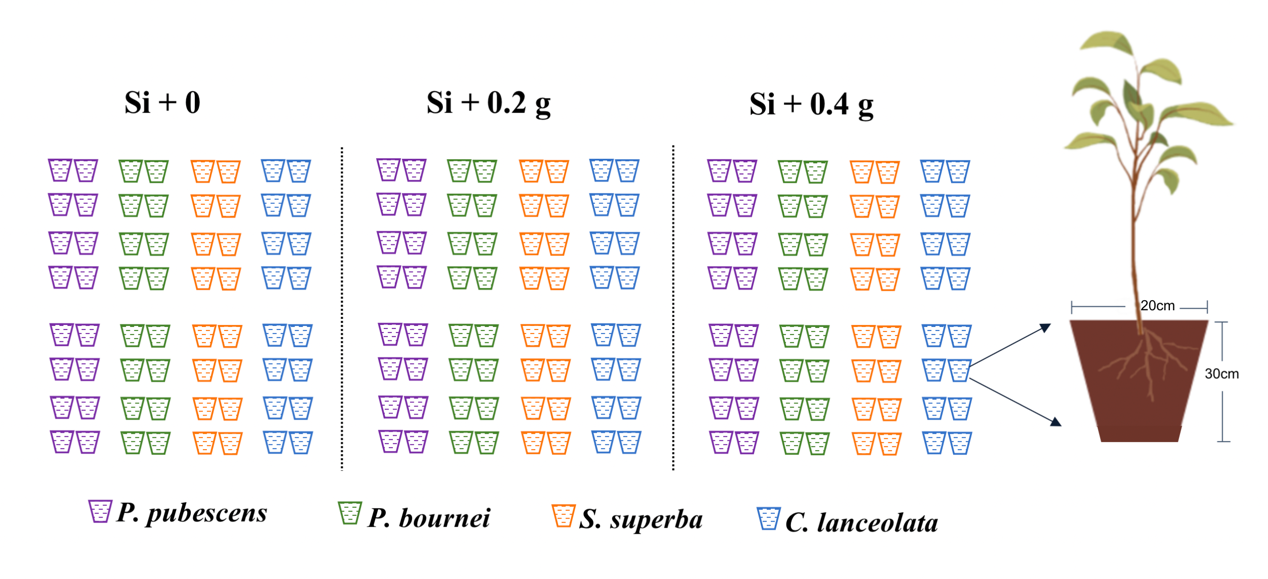 |
| --- |
| **Figure S1. Conceptual diagram of the pot experiment, including three levels of Si supply:** 0 (control), 0.2 g, and 0.4 g Si addition per pot. |

| 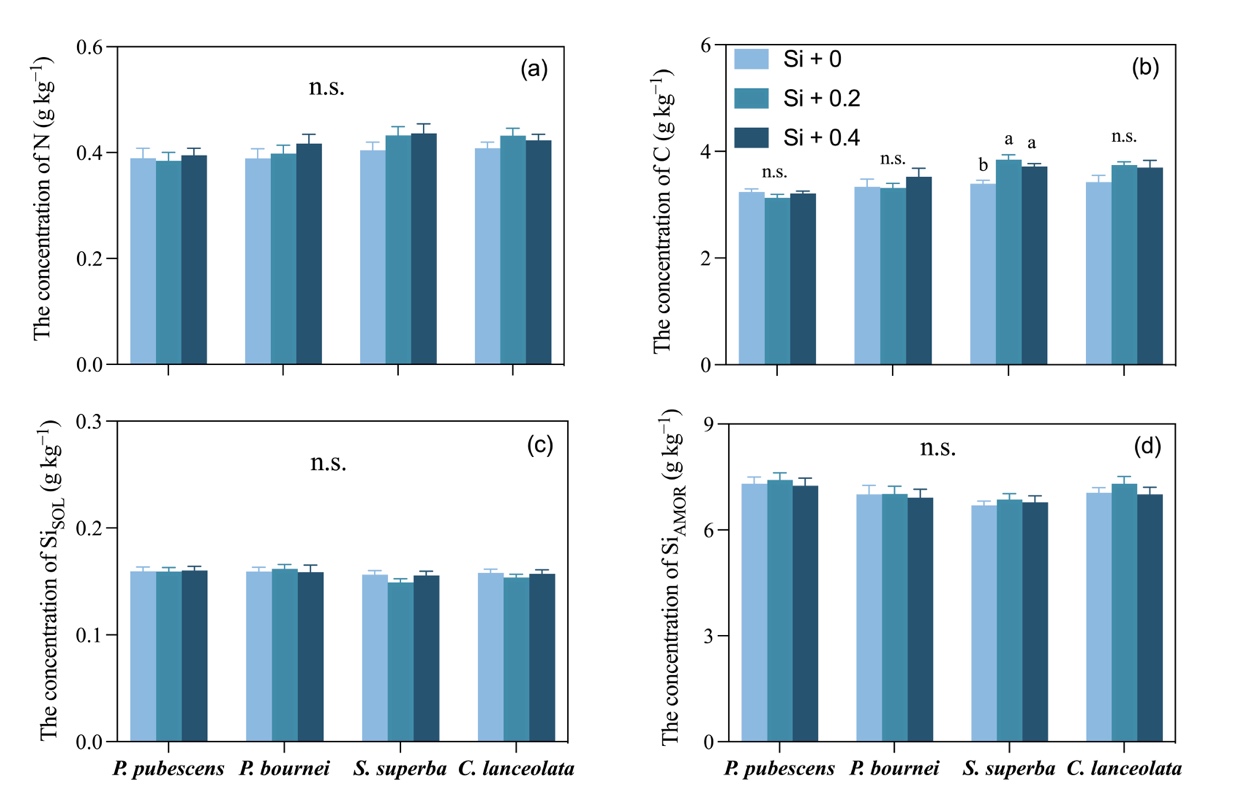 |
| --- |
| **Figure S2. The concentration of nitrogen (N,** **g kg^−1^), carbon (C, g kg^−1^), solubl****e** **silicon (****Si_SOL_****,** **g kg^−1^)****, and amorphous silicon (****Si_AMOR_, g kg^−1^) in soils under three levels of silicon supply.** Error bars depict means with standard errors (*n* = 8). Different letters above bars reflect significant differences among groups (*P* < 0.05). |
